# The moderating role of spirituality and gender in Canadian and Iranian emerging adolescents’ theory of mind and prosocial behavior

**DOI:** 10.3389/fpsyg.2023.1134826

**Published:** 2023-03-27

**Authors:** Nadia Khalili, Sandra Bosacki, Victoria Talwar

**Affiliations:** ^1^Department of Educational and Counselling Psychology, McGill University, Montreal, QC, Canada; ^2^Department of Educational Studies, Brock University, St. Catharines, ON, Canada

**Keywords:** theory of mind, prosociality, spirituality, culture, gender

## Abstract

**Introduction:**

While research has found a link between ToM and prosociality in terms of caring and helping others which may also vary across cultures, the moderating role of spirituality and culture of this association in emerging adolescence has received little attention.

**Methods:**

The current study empirically “examined” the role of spirituality and gender in relation to ToM and prosocial behavior in Canadian and Iranian emerging adolescents. A total of 300 (153 girls) emerging adolescents (*M* = 11.502, SD = 2.228) were recruited from Montreal, Canada and Karaj, Iran. A series of double moderation analysis and ANOVA was conducted.

**Results and discussion:**

Results indicated the difference between direct and indirect influences of ToM and its interactions with culture, gender, and spirituality on prosocial behavior. This implies an emerging complex framework which suggests the dynamic nonlinear interactions between these factors. Implications for youth’s social-emotional understanding will be discussed.

## Introduction

This study examined the potential moderating role of gender, spirituality and culture in the link between Theory of Mind (ToM) and emerging adolescents’ prosociality. ToM refers to mental abilities to understand and explain others’ minds and predict their behaviors (Peterson and Wellman, 2019; Lecce and Devine, 2021), and prosocial behavior refers to voluntary actions that benefit others (Eisenberg, 2003; Imuta et al., 2016). According to theoretical studies, ToM and prosocial development reciprocally interact with each other (Weller and Lagattuta, 2014; Lane and Bowman, 2021), while empirical studies have yielded inconsistent results (Imuta et al., 2016). Furthermore, although earlier research suggests that social and contextual factors such as spirituality and culture may play a moderating role between prosocial and theory of mind abilities (Wang et al., 2022), this has rarely been examined. Thus, the current study aimed to explore the moderating role of spirituality and gender in the relations between ToM and prosocial behavior in Canadian and Iranian youth.

## ToM development and prosocial behaviors in children and adolescents

Theory of mind ability refers to the inference and reasoning of others’ mental states such as intention, belief, desire, and emotion (Peterson and Wellman, 2019), and plays a key role in a person’s social life. Studies with youth show that ToM has a reciprocal interaction with numerous social–emotional variables (Razza and Blair, 2009; Shakoor et al., 2011; Slaughter et al., 2015; Białecka-Pikul et al., 2017) such as children’s friendship (Hughes, 2011; Bosacki, 2021; Gazelle et al., 2022), popularity (Slaughter et al., 2015), social competence (Wainryb and Brehl, 2006; Devine and Apperly, 2022), and moral development (Hughes and Leekam, 2004; Leslie et al., 2006). For example, studies show that those youth who are challenged in their ability to mentalize or read another person’s mind may also be at risk for social well-being and social relationships (Bagwell et al., 1998; Hughes and Leekam, 2004). However, empirical findings suggest that ToM has a complex relationship with social communications, such as prosocial behavior (Hughes and Leekam, 2004; Derksen et al., 2018). For instance, children with advanced ToM abilities have the potential for both antisocial and prosocial behavior (Hughes and Leekam, 2004; Imuta et al., 2016; Derksen et al., 2018). Prosocial behavior is one of the crucial aspects of children’s social competency, which develops from infancy to adulthood from simple concrete assistance to complex abstract supports (Warneken and Tomasello, 2006, 2007; Hastings et al., 2015).

According to earlier studies understanding others’ mental states such as needs, desires, emotions, thoughts, and intentions eases and promotes children’s prosocial development, which further advances their ToM abilities (Eisenberg and Fabes, 1998; Astington et al., 2002; Sy et al., 2003; Hay and Cook, 2007; Weller and Lagattuta, 2014). There have been theoretical links made about the reciprocal interactions between ToM development and prosocial behavior. According to social-construct theory, children develop and strengthen their ToM skills through their social interactions (Carpendale and Lewis, 2015). For example, studies show high levels of prosocial behaviors (sharing, comforting, cooperating) relate to sophisticated ToM skills (Eisenberg and Fabes, 1998; Astington, 2003; Weller and Lagattuta, 2014). Alternatively, some have argued that understanding others’ desires, feelings, and intentions facilitates children’s ability to engage in prosocial behavior toward others (Hoffman, 2000; Hay and Cook, 2007; Dunfield, 2014).

According to Imuta et al. (2016), despite the theoretical justification of reciprocal interaction between ToM development and prosocial behavior, empirical studies have illustrated inconsistencies in this relationship. That is, empirical studies show inconsistencies in the directions of inter-relations between ToM and prosocial behaviors (Imuta et al., 2016; Lane and Bowman, 2021). For example, Hughes and Leekam (2004) and Derksen et al. (2018) suggested that ToM development could positively, negatively, or neutrally influence and be influenced by social relationships. Underwood and Moore (1982) found positive relations between prosocial behavior and 3–13 years old’s affective and cognitive perspective taking. While other studies show that affective ToM (e.g., emotion recognition) has a stronger correlation with prosocial behavior (e.g., sharing and cooperating) rather than cognitive ToM (Carlo et al., 2010; Imuta et al., 2016).

One valid ecological methodology to investigate the complex link between ToM and prosocial behavior is to examine the moderators of this association. A few studies suggested age and gender moderate the relations between ToM and antisocial behavior (e.g., Gomez-Garibello and Talwar, 2015; Mizokawa and Hamana, 2020). Yet to our knowledge, only one study to date has examined the moderating role of gender on the associations between ToM and prosocial behavior in Italian children (Longobardi et al., 2019). Cross-cultural research in this domain (social cognition and prosocial behaviors) continues to remain sparse, especially with young adolescents (Chen et al., 2021; Wang et al., 2022).

### Spirituality

Exploring factors that moderate this relation may resolve the inconsistencies found between ToM and prosocial behavior. One neglected social–emotional factor that potentially moderates the relation between ToM and prosocial behavior is spirituality. Both theoretical and empirical studies suggest that spirituality is centred on meaningful and conscious social relationships, which result in prosocial behavior (Hardy and Carlo, 2005a,b; Pandya, 2017). These studies found that spiritual people value acts of collective compassion such as helping, serving, and caring for others, which are prosocial behaviors (Hardy and Carlo, 2005a,b; Pandya, 2017). To date, only a few studies examined the relation between ToM and spirituality (e.g., Van Elk and Aleman, 2017; Testoni et al., 2019). These studies found a link between the ToM network in brain and different aspect of spirituality such as serving, thinking about God, and praying. Thus, such studies suggest that the ability to enact spirituality such as caring for and helping others requires ToM or the ability understand others’ thoughts and emotions (Barrett, 2004).

However, diverse types of spirituality emphasize various kinds of relationships. For example, existential spirituality emphasizes internal or emotional strength and development (Post and Wade, 2009), while religious spirituality focuses on external development such as institutional and religious community engagement (Spilka et al., 2003; Vittengl, 2018). Religious spirituality can measure either individual relationships with God or social relationships. In contrast, existential spirituality is not necessarily related to social relationships, it can be considered a mental state shaped by social influences (Saunders and Fernyhough, 2016).

Despite numerous studies on spirituality and prosocial behaviors, as well as ToM and prosocial behaviors, to date, no study examines the relations among these three factors. Furthermore, because previous studies evaluated the above relationships with social-based spirituality, exploring the link between individual emotional spirituality, ToM, and prosocial behavior is necessary to understand the relationship between spirituality and ToM more comprehensively. In this research, both religious and existential spirituality were measured as states of being and feeling spiritual, which were not examined in previous studies.

### Culture

Culture is another potential moderator on the relation between ToM and prosocial behavior that has received little attention. Past studies showed mixed findings regarding the influence of culture on ToM, and prosocial behavior separately, while both are key factors in children’s well-being, health, and social relationships (Martí-Vilar et al., 2019; Souza et al., 2021). Thus, discovering the links between cultures and the above social emotional factors is vital to advancing our understanding of how culture affects youths’ mentalization skills and their social relationships.

Culture, however, is not a monolithic phenomenon. Theoretical explanations of culture consider the differences between the various cultures of the world. In the social sciences, most cross-cultural studies have focused on the contrast between North America and China as a prime example of Western individualism versus Eastern collectivism (Chen et al., 2021, 2022). However, studies of the Middle East can also contribute to the understanding of collectivist cultures. In contrast to East Asian countries such as China, Middle Eastern countries’ political and cultural constructions have been influenced by Islam (Rabiei, 2013; Rezapour et al., 2019). Linguistic diversity and political conflicts in the Middle East also have shaped socio-cultural differences within this region (Rabiei, 2013).

Therefore, collectivistic roots and values in the Middle East are notably different from those in other parts of Asia (Shahaeian, 2015; Shohoudi Mojdehi et al., 2020). As one of the world’s oldest civilizations in this area (Barrington, 2012), Iran has experienced several changes in religion, language, and political structure throughout its history (Abrahamian, 2021). Thus, research from this area has a specific context and could contribute to cross-cultural studies by illuminating the cultural differences in social–emotional development in emerging adolescents. Thus, a comparison of data in Canadian and Iranian culture could yield wider insights about the cultural influences on social emotional development, clarifying the relative benefits and challenges of monocultural versus multicultural contexts.

ToM abilities and prosocial behavior occur within a social context that partly depends on the communication styles valued in diverse cultures. Collectivist cultures such as East Asian and Middle Eastern countries follow a high-context communication style, while individualist cultures such as North America follow a low-context communication style (Chitakornkijsil, 2010; Mojdehi et al., 2020). High-context communications emphasize information and usually entail ambiguity and indirect messages, which can be understood through nonverbal actions such as gestures that require little or no speech or text (Nishimura et al., 2008; Mojdehi et al., 2020). Word choice and context are thus important to convey deep meanings through short sentences or words. In contrast, low-context communications emphasize direct speech and literal meaning with the use of precise words that do not depend on the context to be understood (Gudykunst et al., 1988; Mojdehi et al., 2020).

### Gender

Only few studies examined the gender as a moderator between ToM and prosocial behavior (Smith and McSweeney, 2007; Bosacki et al., 2020), however to date, no study has yet to examine the interactions among adolescents’ gender, spirituality, ToM and prosociality between cultures. According to gender socialization theory, boys and girls behave differently in social situations because of their different nurturing practices and experiences (Leaper and Farkas, 2015). Some empirical studies provided evidence for this theory by associating girls with caring behaviors and boys with competitive and assertive behaviors (Kuhnert et al., 2017; Quenneville et al., 2022).

However, inconsistencies in past studies necessitate further investigations in this area. For example, Hughes et al. (2011) found no significant differences in boys’ and girls’ ToM understanding, whereas Białecka-Pikul et al. (2017) and Bosacki (2000) suggested girls are more advanced in these abilities. According to three studies (Longobardi et al., 2016, 2019; Van der Graaff et al., 2018), girls scored higher in prosocial behaviors, while four other studies (Roberts and Strayer, 1996; McMahon et al., 2006; Braza et al., 2009; Longobardi et al., 2016) suggested stronger prosocial behaviors in boys. The present study contributes to the literature by investigating how gender and its interaction with spirituality may moderate the relation between ToM and prosocial behavior across two cultures (Canada and Iran).

## Current study

Few previous ToM studies have focused on emerging adolescence, which is a sensitive transitional period from childhood to adolescence, including significant social, emotional, and cognitive changes that shape young people’s identities (Crocetti, 2017; Bosacki et al., 2018). With the increase of gender-role stereotypes and peer interactions during this transition, emerging adolescents have reciprocal and complex interactions with culture and other social context such as spirituality (Bosacki and Moore, 2004; Andrews et al., 2021).

Considering this context, the current study examines the links between culture, gender, ToM, prosocial behavior, and spirituality in emerging adolescents. Previous research has mainly investigated the association between cognitive ToM (i.e., the ability to make inferences about others’ thoughts and beliefs) and prosocial behavior, recent studies, however, have investigated this link with affective ToM (i.e., the ability to understand others’ emotional states) which may be more strongly correlated with prosocial behavior (Imuta et al., 2016). In this study, to measure ToM, we use the Reading the Mind in the Eyes (RME) test, which evaluates various mind states (e.g., skeptical, accusing, anticipating, reflective, worried, upset, serious, and nervous) which, includes both cognitive and affective ToM (Baron-Cohen et al., 2001; Prevost et al., 2014).

To explores whether culture, gender, and spirituality moderate the relations between ToM and prosocial behaviors in emerging adolescents, this study answers three questions: (1) Do ToM, prosocial behavior and spirituality differ across gender and culture? If so, (2) do gender/culture/spirituality serve as moderators in the relation among ToM, prosocial behavior, (3) What direct and indirect influences do ToM ability have on prosocial behavior in emerging adolescents?

Based on past studies that show gender and cultural differences in prosocial behavior, and spirituality among emerging adolescents (Renouf et al., 2010; Bosacki et al., 2018; Aival-Naveh et al., 2019), the present study also predicts that culture and gender differences influence these variables. We also hypothesize that girls will perform higher on ToM, prosocial behavior, and spirituality (Bosacki, 2000; Leaper and Farkas, 2015; Białecka-Pikul et al., 2017; Wang et al., 2022). Furthermore, given the mixed findings of past studies on the direct and indirect influence of ToM on prosocial behavior (Lane and Bowman, 2021), we expected the same conclusions. Therefore, we hypothesized an emerging complex framework which suggest the dynamic interaction between components that influence ToM and prosociality. According to this framework, relationships between ToM and prosocial behavior vary in contextual conditions. (Gershkoff-Stowe and Thelen, 2004; Blijd-Hoogewys and van Geert, 2017).

## Methods

### Participants

A total of 300 emerging adolescents between 10 and 12 years of age were recruited from Canada and Iran. Canadian adolescent participants (*n* = 150; 78 females; *M* years = 11.502, SD = 2.228) were through schools in Canada. Iranian participants (*n* = 150, 75 females, *M* years = 11.502, SD = 2.228) were recruited for participation in this study through schools in Karaj, Iran.

### Materials

#### Spiritual well-being

The spiritual well-being scale measures both religious and existential well-being, including sense of purpose and meaning in life. The 20-item measure uses a 6-point Likert-type scale ranging from 1 (strongly agree) to 6 (strongly disagree). For the overall scale, scores range from 20 to 120 points; higher scores indicating greater levels of spiritual well-being. Internal consistency coefficients range from 0.82 to 0.94 for the religious well-being subscale, 0.78 to 0.86 for the existential well-being subscale, and from 0.89 to 0.94 for the whole scale. Reliability between 1st and 10th week of testing ranged from 0.82 to 0.99 (Bufford et al., 1991).

#### Children spiritual lives

This questionnaire is a self-report, 31-item scale, developed by Moore et al. (2016) based on a previous qualitative study by Moore et al. (2012). The questionnaire is designed for students from different religious and cultural backgrounds, specifically in North America (see Moore et al., 2016). This measure examines three main factors in relation to spirituality: comfort that “focus on God as a source of support and comfort”, omnipresence that “concerns the ubiquity of God” and duality, a believe that we have a spirit apart from body” (Khalili et al., 2022, p. 30). The participants were asked to respond to items using a Likertscale, between 1 (strongly disagree) and 5 (strongly agree). Inter reliability of this questionnaire was reported between 0.80 and 1.00 on all interviews (Moore et al., 2012).

#### Reading the mind in the eye test third edition

To measure affective ToM, we used the RMET. Past studies show adequate internal consistency for this frequently used measurement with children and youth (α = 0.86; Baron-Cohen et al., 2001; Caputi et al., 2018). The questionnaire includes 36 items, each item contains a picture of an expression with the eyes with four different words indicating four different emotions. The participant should choose the word that best describe the expression. Each item has one correct answer with one point. A higher score indicated a higher ability of reading others emotion.

#### Prosocial behavior

To measure prosocial behavior, we used teacher ratings. In particular, we used a subscale of Children’s Social Behavior Scale (CSBS) – Teacher-Rated, including 4 items, used for measuring prosocial behavior. CSBS is a five-point Likert at scale1 = this is never true of this child, to 5 = this is almost always true of this child. This 15-item survey has three subscales: relational aggression, physical aggression, and prosocial behaviors which measure children’s behaviors with their peers through teachers (Denham, 1986).

### Procedure

Upon obtainment of ethical clearance from the participating universities and school boards, informed letters of consent were sent to principals, teachers, parents, and students. Once written and informed parental consent, and child assent were obtained, self-report pencil-and-paper tasks were administered by the research team within classrooms, or within an alternate room in the school.

## Result

### Preliminary analyses

To investigate the moderating role of spirituality, gender, and culture in the link between ToM and prosociality, we conducted a series of double moderation using R studio. Results indicated that spirituality, gender, culture, and their interaction moderate the association between ToM and prosociality. Data clearing involved the exclusion of 6 participants from Canada, and 20 from Iran, who did not indicate their gender, and 54 Iranian participants who completed less than 50% of the questions. We also removed four outliers from the Canadian dataset. The final sample consisted of 143 Canadian children, and 78 Iranian children (*F* = 120, *M* = 101). Normality, additivity, and homogeneity assumptions were checked for the remaining 216 participants; no significant violence was found in assumption except normalcy of prosocial behavior which does not influence our analysis.

### Descriptive analysis and ANOVA

Descriptive analysis in Table 1 indicates means and standard deviations for ToM (RME), prosocial behavior, spiritual well-being subscales, and children’s spiritual lives subscales. To investigate the cultural and gender effect on our participants’ perception of spirituality, ToM, and prosocial behavior, we conducted a series of univariate analyses of variance (two-way ANOVA) that included gender (female/male) and culture (Canada/Iran) as independent variables. ToM, prosocial behavior, existential well-being, religious well-being, omnipresence, comfort, and duality were the dependent variables.

**Table 1:** Descriptive statistics.

	ToM	Prosocial behavior	Comfort	Omnipresence	Duality	Religious well-being	Existential well-being
*N*	221	221	221	221	221	221	221
Mean	17.08	16.06	2.83	3.21	3.44	4.02	4.47
Std. Deviation	4.276	5.118	1.128	1.124	1.320	1.217	0.761

Independent observations, normality, and homogeneity assumptions were met. Participants were randomly recruited without any interaction to affect each other’s answers, and each record represents a distinct person; thus, observations were independent. A normality check is needed for a small sample size, *N* < 25 per subgroup; Since the sample contained 221 participants with four groups (girls/boys/Canadian/Iranian subgroups) (4*25 = 100), we assumed there was no violation of the normality assumption. Lastly, because our sample sizes were not equal in gender, homogeneity was tested by running Levene’s Test of Equality of Error Variances. Levene’s Test was not significant for the effect of culture and gender on any of variables except for omnipresence (*p* = 0.004). Therefore, we ran the Welch unequal variances test, to determine whether the different group sized impacted homogeneity assumption for omnipresence as indicated by Levene’s test, *F* (1, 217.581) = 4.926, *p* < 0.05. Therefore, our ANOVAs were robust, and statistical assumptions were met.

### Cultural effects

A main effect for culture was found for all variables (see Table 2 for descriptives): ToM abilities, *F* (1,217) = 37.558, *p* < 0.001, $\eta^{2}$ =0.148; Prosocial behavior, *F* (1,217) = 7.966, *p* < 0.01, $\eta^{2}$ =0.035; Comfort, *F* (1,217) = 64.645, *p* < 0.001, $\eta^{2}$ *=0.230;* Omnipresence, *F* (1,217) = 54.815, *p* < 0.001, $\eta^{2}$ =0.202; Duality *F* (1,217) = 84.755, *p* < 0.001, $\eta^{2}$ *=0*.279*;* Religious well-being, *F* (1,217) = 12.519, *p* < 0.001, $\eta^{2}$ =0.055; And existential well-being *F* (1,217) = 49.384, *p* < 0.001, $\eta^{2}$ =0.185 (see Table 2 for descriptives)*.*

**Table 2:** Descriptive statistics of subgroups divided by culture & gender.

	ToM	Prosocial behavior	Comfort	Omnipresence	Duality	Religious well-being	Existential well-being
Canada: Mean (SD)	18.17(0.32)	16.75 (0.42)	3.22 (0.08)	3.56 (0.08)	3.93 (0.10)	3.82 (0.10)	4.72 (0.06)
Iran: Mean (SD)	14.81(0.44)	14.75(0.57)	2.10(11)	2.52(0.11)	2.48(0.13)	4.41(0.14)	4.05(0.08)
Female: Mean (SD)	17.61(0.37)	15.94(0.48)	2.76(0.09)	3.23(0.10)	3.44(0.11)	4.12(0.11)	4.27(0.06)
Male: Mean (SD)	15.37(0.41)	15.55(0.53)	2.54(11)	2.85(0.11)	2.97(0.12)	4.11(0.13)	4.50(0.07)

### Gender effects

A significant main effect of gender was found for ToM abilities (*F* (1,217) = 16.815, *p* < 0.01, $\eta^{2}$ *=0*.072); *Omnipresence* (*F* (1,217) = 7.238, *p* < 0.01, $\eta^{2}$ =0.032); Duality (*F* (1,217) = 8.675, *p* < 0.01, $\eta^{2}$ =0.038); *Existential* well-being (*F* (1,217) = 5.631, *p* < 0.05, $\eta^{2}$ =0.025). However, there was no significant main effect of gender on religious well-being (*F* (1,217) = 0.002, *p* = 0.960, $\eta^{2}$ =0.000); Prosocial behavior (*F* (1,217) = 2.770, *p* = 0.302, $\eta^{2}$ =0.001); And comfort (*F* (1,217) = 2.488, *p* = 0.116, $\eta^{2}$ =0.001) (see Table 2 for descriptives)*.*

### Culture*gender effects

The interaction of culture and gender has a significant effect on ToM (*F* (1,277) = 5.771, *p* < 0.05, $\eta^{2}$ =0.026); Religious well-being (*F* (1,217) = 3.965, *p* < 0.018); And existential well-being (*F* (1,217) = 7.209, *p* < 0.01, $\eta^{2}$ =0.032)*.* However there was no significant influence of the interaction of culture and gender on prosocial behavior (*F*(1,217) = 2.572, *p* = 0.110, $\eta^{2}$ =0.012); Comfort (*F* (1,217) = 0.078, *p* = 0.780, $\eta^{2}$ =0.000); Omnipresence (*F* (1,217) = 008, *p* = 927); And duality (*F* (1,217) = 0.499, *p* = 481, $\eta^{2}$ =0.002) (see Table 3 for descriptives).

**Table 3:** Descriptive statistics of gender*culture.

		ToM	Prosocial behavior	Comfort	Omnipresence	Duality	Religious well-being	Existential well-being
Female	Canada Mean (SD)	18.63 (0.44)	17.51 (0.58)	3.31 (0.11)	3.75 (0.11)	4.10 (0.13)	3.99 (0.14)	4.74 (0.08)
	Iran Mean (SD)	16.59 (0.58)	14.37 (0.76)	2.22 (0.15)	2.71 (0.15)	2.77 (0.17)	4.25 (0.18)	3.81 (0.10)
Male	Canada Mean (SD)	17.70 (0.47)	15.99 (0.61)	3.12 (12)	3.38 (0.12)	3.75 (0.14)	3.65 (0.15)	4.71 (0.08)
	Iran Mean (SD)	13.03 (0.66)	15.12 (0.86)	1.95 (0.17)	2.32 (0.17)	2.19 (0.19)	4.57 (0.20)	4.29 (0.12)

### Moderating role of culture, gender, and spirituality

To determine whether the relation between ToM and prosocial behavior was moderated by culture, gender, and spirituality, we conducted a series of double moderations. As moderating variables could change the magnitude or the direction of the relations between IVs and DVs, we tested whether these moderators would either strengthen or weaken the effect of the ToM on the prosocial behavior; and if so, whether in the positive or inverse direction. For the purpose of the moderating analysis, gender was dummy coded, and all continuous variables were mean centralized. Therefore, the coefficient of IVs and moderators will be interpreted as the effect of these variables on DV at the mean level of the other independent variables.

#### Spirituality and gender

To examine the moderating role of spirituality and gender on the relations between ToM and prosocial behavior, we conducted several double moderations using R studio and packages “devtools” and “doomlab/MeMoBootR.” In all models, ToM was entered as an independent variable (“*X*”), and prosocial behavior entered as a dependent variable (“*Y*”). The interaction of gender with subscales of spiritual well-being and children’s spiritual lives was examined as pair moderators. Only existential well-being*gender, *F* (5,215) = 6.526, *p* < 0.001, $\eta^{2}$ =0.131, and duality*gender, *F* (5,215) = 7.489, *p* < 0.001, $\eta^{2}$ =0.148, showed a significant moderation effect on the relations between ToM and prosocial behaviors which will be discussed below.

#### Existential well-being and gender

When Existential well-being and gender are entered as moderators, Existential well-being (*b* = 6.204, *t*(1.708) = 3.632, *p* < 0.00), while Gender (*b* = 0.265, *t*(0.097) = 2.734, *p* < 0.01), negatively predicts prosocial behavior and ToM (*b* = 1.263, *t*(0.454) = 2.782, *p* < 0.01) positively predict prosocial behavior. Moderation effects of the interaction between IVs and Ms. such as ToM*existential well-being (*b* = −0.265, *t*(0.097) = −2.734, *p* < 0.01) and ToM*gender (b = −0.486, *t*(0.157) = −3.096, *p* < 0.01) negatively predict prosocial behavior (see Table 4). In other words, existential well-being negatively influences and weakens the impact of ToM on prosocial behavior. Furthermore, the relation between ToM and prosocial behavior was different in males and females. Gender served as a moderating variable as results showed that ToM had a negative influence on prosocial behaviors in males, but not in females. That is, males with high levels of ToM were more likely to demonstrate low levels of prosocial behaviors.

**Table 4:** Moderating role of existential well-being and gender on the relationship between ToM and prosocial behaviour.

Existential well-being* gender	*b*	SE	*t*	*p*
Existential well-being	6.204	1.708	3.632	0.000
Gender	−0.265	0.097	−2.734	0.011
ToM	1.263	0.454	2.782	0.005
ToM* existential well-being	−0.265	0.097	−2.734	0.006
ToM* gender	−0.486	0.157	−3.096	0.002

The overall model shows that all individual predictors, ToM, existential, gender, ToM*existential well-being, and ToM* gender, together predict 13.1% of the variance in prosocial behavior. The interaction of ToM and existential well-being contributes to a 3% variance of prosocial behavior in this model. The interaction of ToM and gender contributes to a 3.8% variance of prosocial behavior in this mode. Both interactions together contribute to a 6.3% of variances in prosocial behavior.

**Figure 1:**
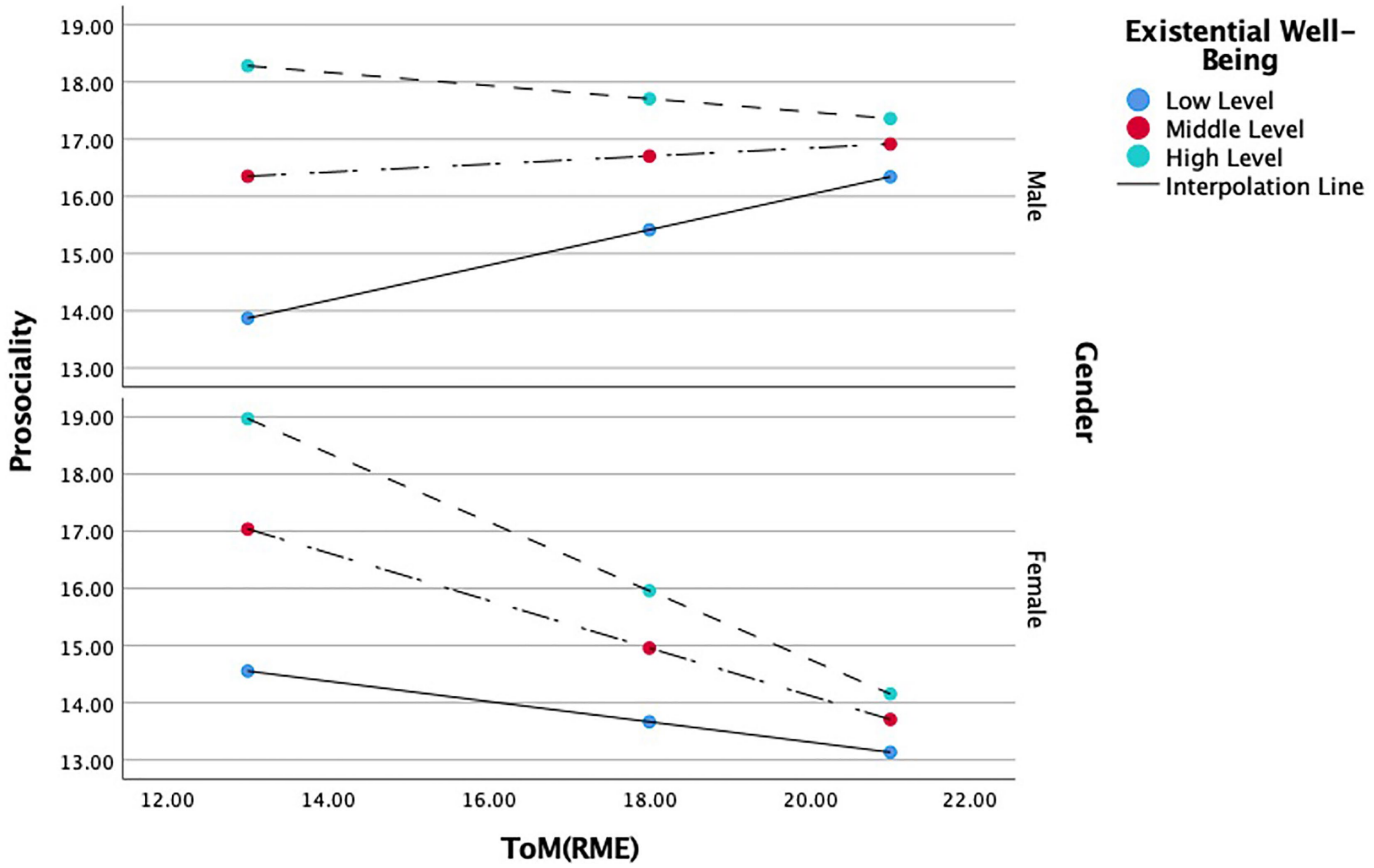
Moderating role of existential well-being and gender in the relationship between ToM and prosociality.

The conditional effects of the focal variables show that a high level of existential well-being had a strong negative moderating influence on the relations between female ToM and prosocial behavior (*b* = −0.601, *t*(0.140) = −4.289, *p* < 0.001), while no significant moderation effect was found for males. That is, high levels of existential well-being related to high ToM, and low levels of prosocial behavior. In contrast, low levels of ToM predicted high levels of prosocial behavior, but *only* in girls. A moderate level of existential well-being has a moderate negative effect on the relations between female ToM and prosocial behaviors (*b* = −0.416, *t*(0.118) = −3.503, *p* < 0.001), *whereas* no significant moderation effect was found for males. Accordingly, moderate level of existential well-being related to higher levels of ToM and in turn, predicted low prosocial behavior. In contrast, low levels of ToM predicted moderate levels of prosocial behavior, but *only* in girls. Low levels of existential well-being showed a moderate positive influence on the relation between ToM and prosocial behavior in males (*b* = 0.309, *t*(0.140) = 2.205, *p* < 0.05), whereas no significant moderation effect was found for the female group in this model. In other words, boys who reported low levels of existential well-being were also more likely to demonstrate high levels of ToM which in turn predicted moderate levels of prosocial behavior. In contrast, low levels of ToM predicted low levels of prosocial behavior but only in boys (see Figure 1).

**Table 5:** Moderating role of duality and gender on the relationship between ToM and prosocial behaviour.

Duality* gender	*b*	SE	*t*	*p*
Duality	4.531	0.928	4.879	0.000
Gender	9.739	2.781	3.512	0.000
ToM	0.750	0.192	3.893	0.000
ToM* duality	−0.189	0.044	−4.218	0.000
ToM* gender	−0.607	0.159	−3.808	0.000

**Table 6:** Moderating role of culture and gender on the relationship between ToM and prosocial behaviour.

Culture* gender	*b*	SE	*t*	*p*
Culture	−7.284	2.882	−2.527	0.012
Gender	9.244	2.816	3.282	0.001
ToM	−0.544	0.409	−1.329	0.185
ToM* culture	250	0.170	1.470	0.142
ToM* gender	−0.606	0.161	−3.752	0.000

#### Duality and gender

When duality and gender were entered as moderators, ToM (*b* = 0.750, *t*(0.192) = 3.893, *p* < 0.001), duality, (*b* = 4.531, *t*(0.928) = 4.879, *p* < 0.001), and gender (*b* = 9.793, *t*(2.781) = 3.512, *p* < 0.001), positively predicted prosocial behavior. The interaction between ToM and duality (*b* = −0.189, *t*(0.044) = −4.218, *p* < 0.001), and the interaction between ToM and gender, (*b* = −0.607, *t*(0.159) = −3.808, *p* < 0.001), negatively predicted prosocial behavior (see Table 5). In other words, duality weakened the influence of ToM on prosocial behavior, and negatively influenced the relationship between ToM and prosocial behavior. Furthermore, the relations between ToM and prosocial behavior were different amongst males and females.

The overall model showed that all individual predictors, ToM, duality, gender, ToM*duality, and ToM* gender, together predicted *14.8%* of the variance in prosocial behavior. Specifically, the interaction of ToM and duality contributed to a *7%* variance of prosocial behavior. The interaction of ToM and gender contributed to a *5.7%* variance of prosocial behavior in this model. Taken together, both interactions contributed to *10.4%* of variances in prosocial behavior. In other words, the moderators in this model—gender and duality—together contributed to 10.4% variances in outcome.

**Figure 2:**
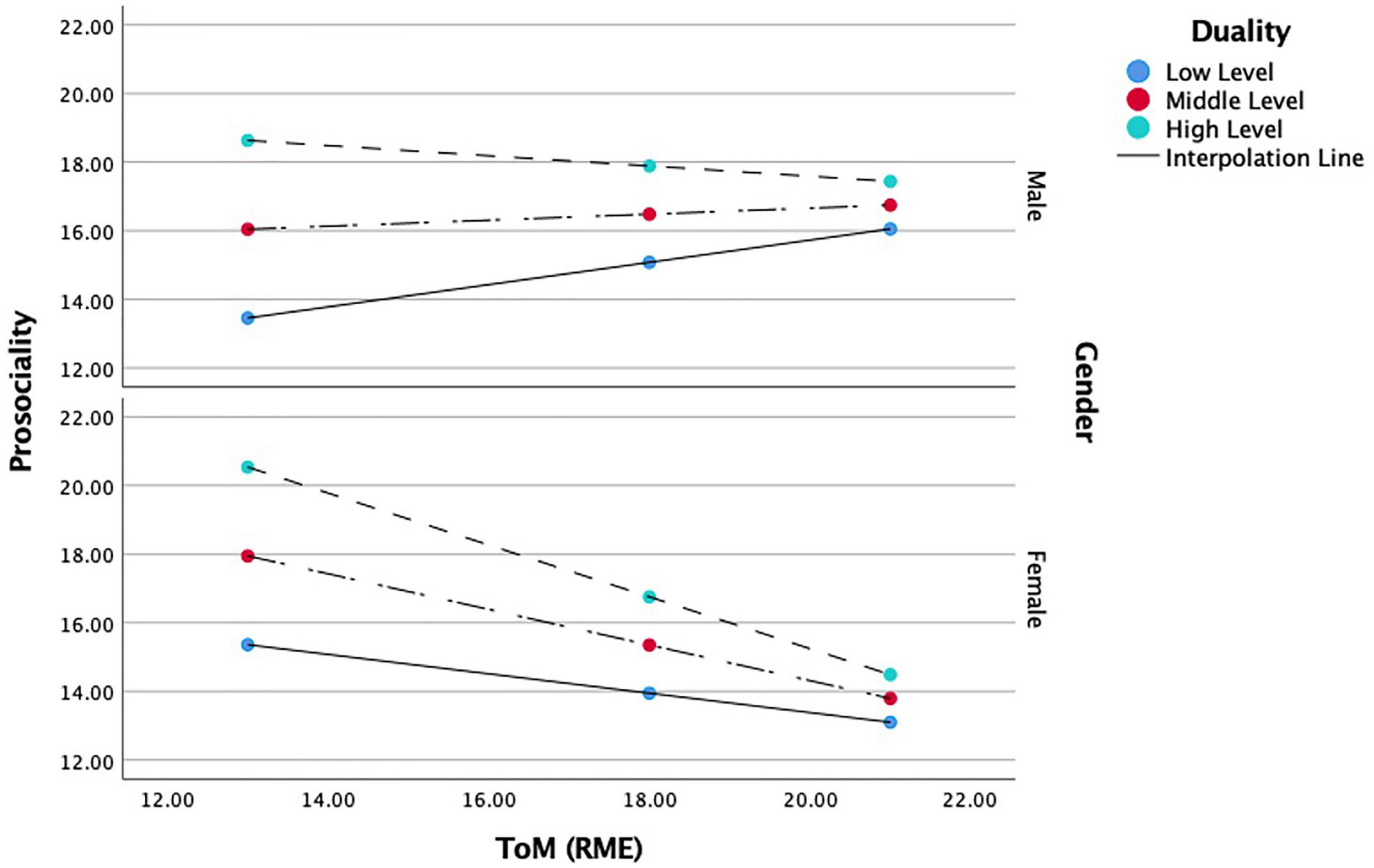
Moderating role of duality and gender in the relationship between ToM and prosociality.

The conditional effects of the focal variables show that a high level of duality has a strong negative moderating influence on the relations between female ToM and prosocial behavior (*b* = −0.755, *t*(0.146) = −5.150, *p* < 0.001) whereas no significant moderation effect was found for the male group in this model. Accordingly, high levels of duality and high levels of ToM predict low levels of prosocial behavior, and high level of duality and low ToM predict high levels of prosocial behavior but only in girls*.* A moderate level of duality has a moderate but statistically significant impact on the negative relation between female ToM and prosocial behaviors (*b* = −0.519, *t*(0.121) = −4.264, *p* < 0.001) whereas no significant moderation effect was found for males. Thus, with moderate levels of duality, high levels of ToM predicted low levels of prosocial behavior, and low levels of ToM predicted moderate prosocial behavior, but again, only in girls. Low levels of duality have a moderate positive influence on the relations between male ToM and prosocial behavior (*b* = 0.324, *t*(0.120) = 2.2683, *p* < 0.01), whereas low levels of duality have a moderate negative influence on the relations between female ToM and prosocial behavior (*b* = −0.300, *t*(0.120) = −2.353, *p* < 0.05). So, with low duality, high ToM predicted moderate levels of prosocial behavior in boys, and low levels of prosocial behavior in girls. However, low ToM predicted low levels of prosocial behavior in boys, and moderate levels of prosocial behavior in girls (see Figure 2).

#### Gender and culture

When gender and culture were entered as moderators, the overall model was significant: *F*(5,215) = 6.169, *p* < 0.001, $\eta^{2}$ =0.125. Gender, (*b* = 9.244, *t*(2.816) = 3.282, *p* < 0.001), positively predicts prosocial behavior. Culture, (*b* = −7.284, *t*(2.882) = −2.527, *p* < 0.05), and the interaction between ToM and gender, (*b* = −0.606, *t*(0.161) = −3.752, *p* < 0.001), negatively predicted prosocial behavior. While ToM, (*b* = −0.544, *t*(0.409) = −1.329, *p* = 0.185), and the interaction between ToM and culture, (*b* = 250, *t*(0.170) = 1.470, *p* = 0.142), was not significant (see Table 6). In other words, gender moderated the relations between ToM and prosocial behavior in this model, whereas culture does not moderate these relations.

**Figure 3:**
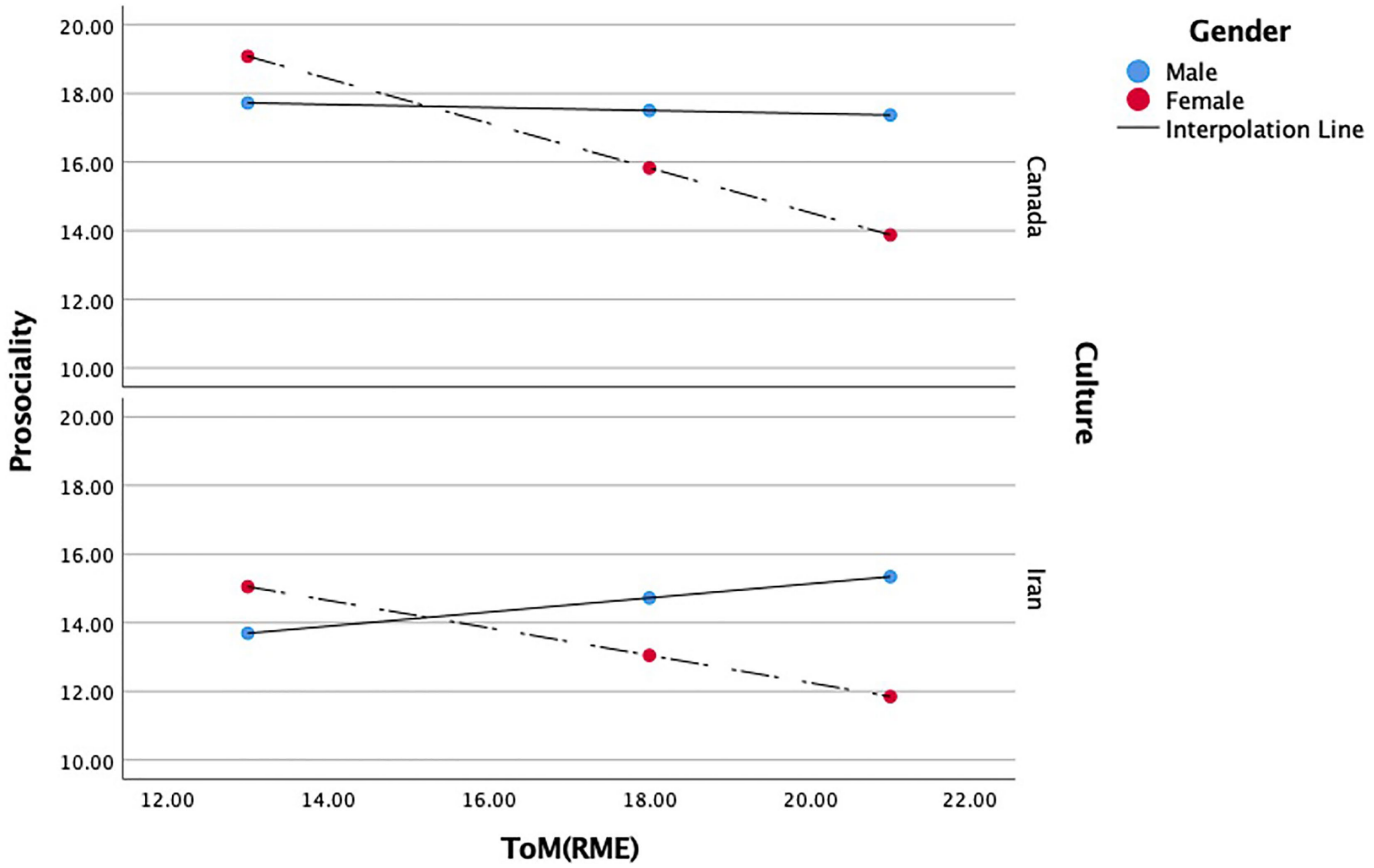
Moderating role of gender and culture in the relationship between ToM and prosociality.

The focal predictors’ conditional effects showed a strong negative relation between Canadian female ToM and prosocial behavior (*b* = −0.650, *t*(0.50) = −4.315, *p* < 0.001), whereas we found no significant effect for males. That is, high ToM predicted moderate levels of prosocial behavior, and low ToM predicted high levels of prosocial behavior only in Canadian females. There was also a moderate negative relation between Iranian female ToM and prosocial behavior (*b* = −0.400, *t*(0.154) = −2.596, *p* < 0.05), whereas no significant effect was found for the males. In other words, high ToM predicted low levels of prosocial behavior, and low ToM will predict moderate levels prosocial behavior only in Iranian females (see Figure 3).

#### Culture and duality

When duality and culture are entered as moderators, the overall model was significant: *F*(5,215) = 4.563, *p* < 0.001, $\eta^{2}$ = 0.095. Duality, (*b* = 3.368, *t*(1.197) = 2.812, *p* < 0.01), positively predicted prosocial behavior. The interaction between ToM and duality, (*b* = −1.390, *t*(0.055) = −2.499, *p* < 0.05), negatively predicted prosocial behavior. While the effect of ToM, (*b* = 0.452, *t*(0.596) = 0.758, *p* = 0.449), culture, (*b* = −0.066, *t*(3.487) = −0.019, *p* = 0.984), and the interaction between ToM and culture: (*b* = −0.062, *t*(0.196) = −3.189, *p* = 0.750), on prosocial behavior were not significant (see Table 7). In other words, compared to culture, duality was the only variable to moderate the relations between ToM and prosocial behavior.

**Figure 4:**
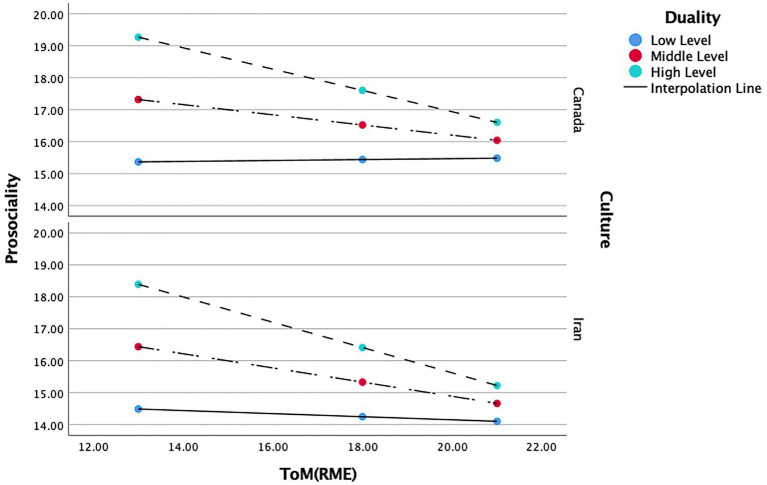
Moderating role of duality and culture in the relationship between ToM and prosociality.

The conditional effects of the focal predictors showed a moderate negative relation between ToM and prosocial behavior (*b* = −0.650, *t*(0.146) = −5.150, *p* < 0.001) in Canadians with a high level of duality, and high negative relation between ToM and prosocial behaviour (*b* = −0.650, *t*(0.146) = −5.150, *p* < 0.001) in Iranians with a high level of duality, while no significant effect was found for other levels of duality in each country (see Figure 4).

#### Culture and existential well-being

When duality and culture were entered as moderators, the overall model was significant: *F*(5,215) = 4.845, *p* < 0.001, $\eta^{2}$ = 0.148. Existential well-being, (*b* = 5.267, *t*(2.059) = 2.557, *p* < 0.05), and the interaction between ToM and existential well-being, (*b* = −0.243, *t*(0.119) = 2.557, *p* < 0.05), positively predicted prosocial behavior. However, ToM, (*b* = 0.970, *t*(0.920) = 1.054, *p* = 0.292), culture, (*b* = −1.551, *t*(3.424) = -0.453, *p* = 0.651), and the interactions between ToM and culture: (*b* = −0.016, *t*(0.206) = −0.082, *p* = 0.934), did not have impact on prosocial behavior (see Table 8). In other words, existential well-being moderated the relation between ToM and prosocial behavior in this model, whereas culture did not serve as a moderator.

**Figure 5:**
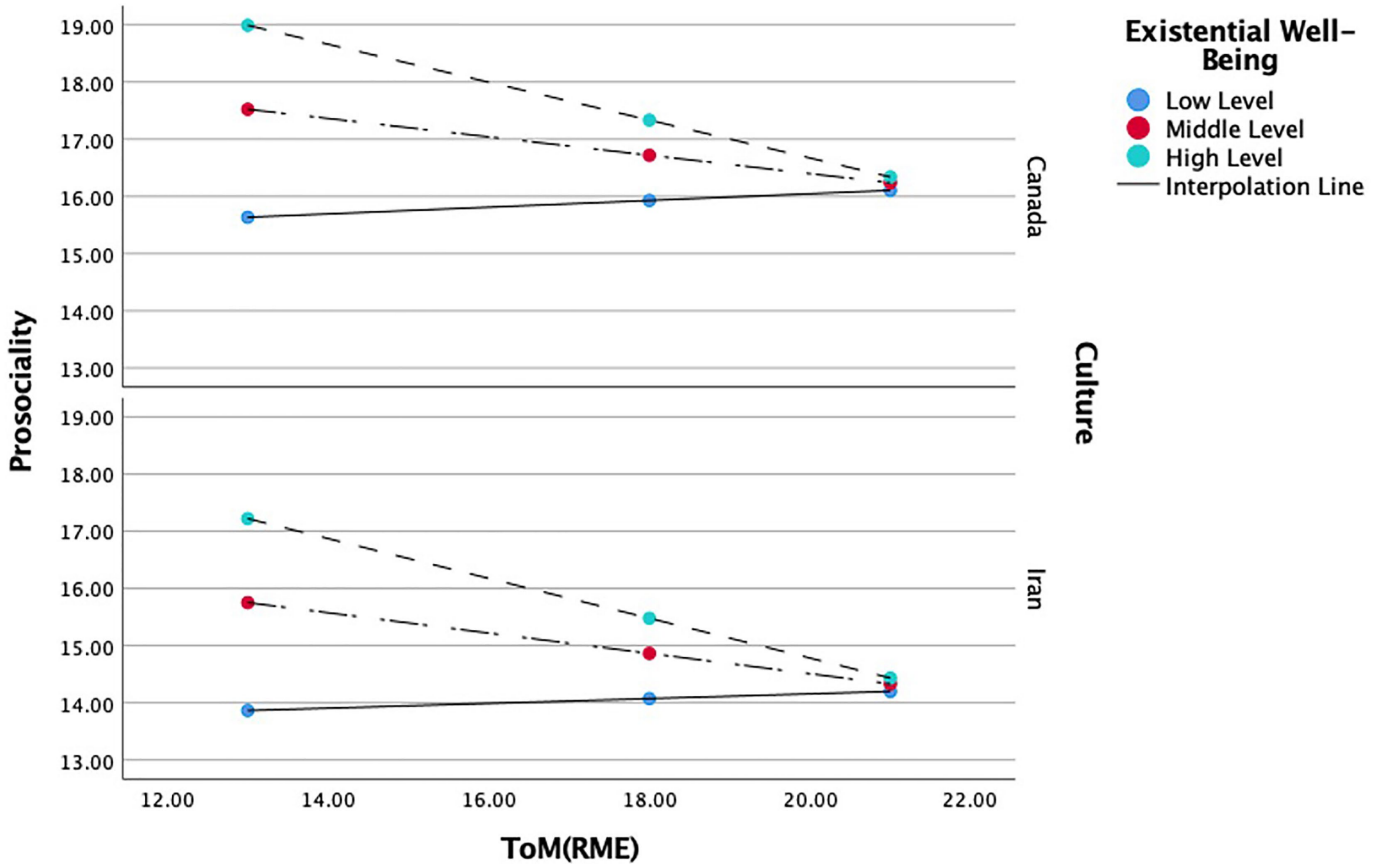
Moderating role of existential well-being and culture in the relationship between ToM and prosociality.

The conditional effects of the focal predictors showed a moderate negative relation between ToM and prosocial behavior (*b* = −0.331, *t*(0.113) = −2.921, *p* < 0.01) in Canadian with a high level of existential well-being, whereas no significant effect was found for other levels of existential well-being on this model. Thus, with high existential well-being, high levels of ToM predicted low levels of prosocial behavior, and low levels of ToM predicted high levels of prosocial behavior (see Figure 5).

## Discussion

The main purpose of this study was to investigate the complex relations between ToM, spirituality, and prosocial development in emerging adolescents in Canada and Iran. More specifically, we investigated the direct and indirect role of emerging adolescents’ ToM ability from two different countries on their prosocial behavior. We also explored if the social-contextual factors of spirituality, culture, and gender served as moderators in the relations between ToM and prosocial behavior. First, we explored the role gender and culture played in emerging adolescents’ prosocial behaviors and their perceptions of spirituality, as well as the ability to recognize emotions in others (affective ToM). Further, to explore the moderating role of gender, culture, and spirituality in emerging adolescents, we investigated the direct and indirect influence of ToM on prosociality.

A key finding from our study was that our results demonstrated significant cultural differences between Canadian and Iranian participants’ ToM ability. These findings suggest the significant differences between understanding others’ thoughts and emotions among Iranian and Canadian youth could be due to differences in sociographic and linguistic factors, parenting styles, and value preferences (Mason and Morris, 2010; Renouf et al., 2010; Aival-Naveh et al., 2019). Compared to Canadian youth, the lower score of ToM in Iranian youth supports past studies that show that in collectivist cultures, teachers and parents are more likely to discourage mental state’ talk in everyday conversations (Adams et al., 2012). In contrast, studies show in Western countries, parent–child conversations are more likely to use mental or internal state language, which in turn may help to strengthen children’s ToM abilities (Adams et al., 2012; Mayer and Tra ¨uble, 2013). Furthermore, high context communication style in countries such as Iran emphasize information using ambiguous and indirect messages that could be understood through context and word choice (Shohoudi Mojdehi et al., 2019). Therefore, the present study’s results suggest that the “reading the mind in the eyes” test could be interpreted differently by Iranian and Canadian youth, due to their different social experience.

The significant differences found in between Iranian and Canadian adolescents’ existential well-being, duality, comfort, and omnipresence, suggests differences in the perception of spirituality among youth in these two countries. Interestingly, Iranian students living in a religious (Islamic) country which declared itself officially Shie and all its institutions follow and teach Islamic laws (Evason, 2016), showed lower scores in existential well-being, duality, and omnipresence, which were found to be highly related to religiosity, compared to Canadian youth who lived within a secular environment. These findings are similar to Hardy et al. (2022) that suggested religious deidentification among British youth and Büssing et al. (2012) that reported a low level of religiosity among Christian adolescents who attended a highly religious school within a strong faith-based community. They interpreted the adolescents’ lower scores as a result of living within a secular society with the unpopularity of religious beliefs.

Within the present study, the present Iranian participants lived within a country lead by a religious government that controlled schools and institutions. We interpret our findings as a sign of dramatic changes in young people’s religious beliefs and secular shifts in Iran due to a critical view of the link between political power and religious authority in Iran (Arab and Maleki, 2020). In line with Arab and Maleki (2020) the current findings provide novel evident to suggest that within the context of governmental and religious pressure, younger Iranian generations are changing their beliefs and attitudes from high religiosity to more anti-religious sentiments. Cognitive resistance and rejection of some religious demands could also be another reason for the low religious and spiritual scores found in the present study’s sample of Iranian youth (Büssing et al., 2012).

The significant differences found between Canadian and Iranian adolescents’ prosocial behaviors suggest cultural differences in their social abilities, such as helping and serving others reported by their parents and teachers. Our study aligns with findings from Luria et al.’s study (2015), that found a slight but positive relation between individualist culture and prosocial behavior. However, our findings contradict those of Parboteeah et al. (2004), and Lampridis and Papastylianou (2017), which showed a positive relation between collectivism and prosocial behavior. On the other hand, Ringov and Zollo (2007) and Onder (2011) found no relations between prosocial behavior and national culture, shared values, behaviors, customs, and norms shared by the population of a certain country (Beugelsdijk and Welzel, 2018; Martí-Vilar et al., 2019). These findings suggest that social–emotional development is complex in nature and could be understood by examining many factors, including national culture.

The interaction between gender and culture showed a significant influence on participants’ ToM ability and existential and religious well-being. In contrast, this interaction between gender and culture failed to have an influence on prosocial behavior, comfort, omnipresence, and duality. Past studies show that compared to boys, girls generally score higher in ToM ability (Spilka et al., 2003; Saunders and Fernyhough, 2016; Vittengl, 2018), and spiritual beliefs regarding omnipresence and duality (Bosacki et al., 2018). However our findings on omnipresence support past studies that show no significant differences in omnipresence between girls and boys (Bosacki et al., 2018). The findings support gender-role socialization theory that suggests gender-role stereotypes encourage girls to demonstrate more nurturing and caring behaviors with others, and thus may be the reason for their higher scores in social–emotional factors such as understanding others’ thoughts and emotions (Eisenberg and Fabes, 1998; Bosacki, 2000; Charman et al., 2002; Leaper and Farkas, 2015; Białecka-Pikul et al., 2017; Wang et al., 2022). Similarly, the present results support past studies that show compared to males, females were more likely to reflect higher levels of religious spirituality – particularly the dimensions of omnipresence and duality which focus on external development and their social relationships and relationships with God (Spilka et al., 2003; Saunders and Fernyhough, 2016; Vittengl, 2018).

In contrast, the present results showed that compared to girls, boys scored higher in existential well-being which could suggest that boys showed a greater tendency to emphasize a sense of self-agency and internal strength development (Post and Wade, 2009). However, some studies show no significant gender differences in ToM abilities and spirituality (Hughes et al., 2011; Khalili et al., 2022), which is also consistent with our findings in that there was no main effect of gender on religious well-being and comfort among participants from both countries. These findings thus support the claims that gender may add to the complexity of the relations between social emotional factors such as ToM and spiritual development in adolescents, particularly across different cultures (Bosacki et al., 2018; Quenneville et al., 2022).

The present results did not show any significant gender differences on prosocial behavior, which is in contrast to previous studies that suggested higher scores of prosociality in girls (Eisenberg et al., 2010; Iglesias Gallego et al., 2020). One justification of these findings could be a connection between gender roles and social–emotional development factors, which aligns with social learning theory – that people learn by observing, imitating, and modelling behaviors in society (Bandura and Walters, 1977; Nabavi, 2012; Jans-Beken et al., 2018; Khalili et al., 2022). Recent studies show the changing nature of societal gender-role expectations (Andrews et al., 2021; Quenneville et al., 2022). For example, Coyne et al. (2021) explored how “princess culture” (in Disney movies and other entertainment) influences gender stereotypes in behavior and body esteem as children learn gender roles through their interaction with their environment, including popular media. These findings demonstrate a generational shift in gender-role stereotypic beliefs and attitudes that may influence children’s socialization, suggesting a need to study how children and adolescents’ social–emotional factors have also been influenced by gender (Gazelle et al., 2022).

In general, the present study found that Canadian girls scored higher in most of the social–emotional factors than boys and Iranian girls, while Iranian boys scored lower for most social–emotional factors (e.g., ToM, prosocial behavior, and spirituality). These findings align with Çelik and Deniz (2008) that girls from Western cultures tend demonstrate stronger abilities in social and emotional competencies compared to girls and boys from other cultures. Furthermore, stronger curriculum concentration on social–emotional well-being and development in Canadian educational systems compared to Iranian educational systems could be another reason for lower scores in Iranian participants (Schonert-Reichl and Hymel, 2007; Ahrari et al., 2022). These cultural and gender variations in our findings suggested the need for studies to explore the moderating role of gender and culture on the indirect influence of adolescents’ ToM on prosocial behavior.

Our moderating analysis suggest that ToM has a direct positive influence on prosocial behavior in both countries, which supports theoretical studies that suggest understanding others’ feelings and thoughts facilitates one’s ability to help and serve others (prosocial behavior) (Hoffman, 2000; Hay and Cook, 2007; Dunfield, 2014). Although many studies (Megías-Robles et al., 2020) refer to “reading the mind in the eyes” as a measurement of affective ToM (understanding others’ feelings), the test evaluates different mind states (e.g., skeptical, accusing, anticipating, reflective, worried, upset, serious, and nervous) which includes both cognitive and affective ToM (Baron-Cohen et al., 2001; Prevost et al., 2014). Therefore, as this test does not categorize these mind states, we conclude that regardless of a children’s culture and level of ToM ability, it is more likely that youth with a higher capacity to understand other’s mental states, both cognitive and affective, show prosocial behaviors.

Our findings support the importance of information expressed in the eye region and its vital link with social interactions (Grossmann, 2017; Schmidtmann et al., 2020). It is also worth mentioning that although the “reading the mind in the eyes” test includes cognitive understanding, it is different from other cognitive tasks such as “False belief understanding,” which are built on reasoning and strongly related to memory, language, and executive function (Guajardo et al., 2009; Diaz and Farrar, 2018). Thus, it is important for future research to examine the influence of children’s ToM ability through different tasks to measure prosocial behavior in everyday life.

Our results showed that indirect influence of ToM on prosocial behaviors was negatively moderated by gender, which was significant in girls but not boys. These findings contradict some past studies that suggest a strong link between the girls’ mindreading abilities and prosocial behaviors (Leaper and Farkas, 2015). We also found that girls with a higher level of ToM ability were less likely to show prosocial behavior while relations were found between boys’ mindreading and prosocial behaviors. Our results specifically contradicts Longobardi et al. (2019) findings that suggested gender positively moderated the relation between ToM and prosocial behavior in Italian children, and emerging adolescents boys and *not* in girls. In other words, their findings suggested no associations between ToM and prosocial behaviors in girls, while our results showed a negative association between these two factors in girls only. However, our results did not show a significant associaton between ToM and prosocial behaviors in boys. These results support neither global patterns nor cultural differences. Still, they suggest individual differences and the complex relationship between social–emotional abilities and the role of contextual conditions such as gender and culture in mentalization skills and spirituality.

Surprisingly, the impact of ToM on prosocial behavior was negatively moderated with spirituality, existential well-being and duality. However, we did not find any moderating role of religious well-being, omnipresence, and comfort in this relationship. The interactions between gender and spirituality showed that girls’ and boys’ different levels of perception of spirituality were associated with various levels of prosociality. Girls with high existential well-being/duality and high ToM showed low prosocial behavior, but no relations were found for boys. Girls with moderate existential well-being/duality and high ToM showed low prosocial behavior, but again not for boys.

Compared to girls, boys with low existential well-being/duality and high ToM showed moderate levels of prosocial behavior. Boys with low existential well-being and low ToM showed low levels of prosocial behavior, but no relations were found for girls. Girls with low duality and high ToM showed low levels of prosocial behavior. Girls with low duality and low ToM showed moderate levels of prosociality. Boys with low existential well-being/duality and high ToM showed average levels of prosocial behavior. These findings contrast with past theoretical and empirical studies that suggest that spirituality is associated with prosocial behavior and ToM ability and suggest a more complex pattern (Barrett, 2004; Hardy and Carlo, 2005a,b; Pandya, 2017; Van Elk and Aleman, 2017; Testoni et al., 2019).

Our findings suggest considering social–emotional development as a complex system which could be understood through a nonlinear analysis. The relations between the components of this system, such as the interconnections among ToM, prosocial behavior, gender, culture, and spirituality, will be better understood through a nonlinear analysis. Nonlinear systems are defined as systems that do not have additivity, and homogeneity are known as the two main characteristics of linear systems (Heeger, 2000; Boissevain and Mitchell, 2018; Tabassum et al., 2018). Additivity means that if we add two components to the system, the result will not be different from merely adding separate elements. Homogeneity states that the strength of the output will change proportionately with the input if we increase the power of the input (Antsaklis and Michel, 2006).

These characteristics imply that how added components to the system interact with each other is more crucial than the number of them. For example, the interaction of two added components could positively, negatively, or neutrally influence the system, which is the opposite of additivity. Increasing the strength of the input also could either decrease or increase the power of input in a system, which is against homogeneity. From a nonlinear perspective, the influence of a factor on other variables cannot be measured separately due to reciprocal interactions among variables (Van Geert, 2003; Lowie, 2012). Our study and findings support this perspective by considering the direct and indirect interactions between different levels of spirituality and prosociality with gender and culture in emerging adolescents’ ToM development.

To elaborate, the results suggest a large difference between Canadians’ and Iranians’ ToM abilities and moderate differences between women’s and men’s ToM abilities. Therefore, from a linear perspective, we should expect a large or medium difference between Canadian females’ ToM ability compared to other participants. Still, our results show a slight difference between Canadian adolescent females’ ToM ability compared to other participants. Considering the positive influence of ToM ability on prosocial behavior, we should expect a moderate or large influence of the interaction of culture and gender on prosocial behavior. However, our results suggest that the interaction between culture and gender did not influence prosocial behavior, which supports the complexity and nonlinearity of ToM development and its relations to other social–emotional factors such as prosociality, culture, and gender.

Our moderating analysis results contrast with Fodor’s (1983) and Parboteeah et al.'s (2004), who discussed the computational nature of social–emotional developments. We suggest a more dynamic and fluid nature of complex nonlinear interactions may help to better untangle the complex links between social-cognitive and social- emotional-cultural factors. By shedding light on the influence of factors such as gender and culture on input, internal structure, and output of a relationship between ToM and prosocial behavior, we propose a shift in perspective (Lowie, 2012).

For example, the influence of gender on ToM, prosocial behavior, and the relations between ToM and prosocial behavior (internal structure) is a moderating model. This model illustrates a moderate gender influence on ToM ability, no gender influence on prosocial behavior, and the negative moderating role of gender on the relationship between ToM and prosocial behavior, while ToM positively predicts prosocial behavior. This suggests a more dynamic and fluid nature of gender influence on the moderating system of ToM and prosocial behavior. Not only does the input (ToM) and output (prosocial behavior) play a role, but the internal structure (the relations between them) is also influenced by gender. Interestingly, these influences are neither linear, nor consistently positive or negative. To elaborate, females showed a high level of ToM ability; and this ToM ability positively predicted prosocial behaviors. Therefore, from a linear perspective, we expected a high level of prosociality in females, while our results showed the opposite- a low level of prosociality in females with a high level of ToM ability.

Overall, this study is novel and contributes to the literature on cross-cultural social cognition, prosociality and spirituality, as this was the first study to examine gender and cultural differences in the relations among mentalization skills, prosocial behaviors and perceptions of spirituality in Canadian and Iranian young adolescents.

Another novel aspect of this study was that it uses a measure of Iranian children perceptions of spirituality, entitled Children Spiritual Lives Questionnaire developed by Moore et al. (2016). Given the lack of research on adolescent’s spiritual understanding within a social cognitive context, this contributes to the cross-cultural literature in the field of young people’s spiritual and psychosocial development. Furthermore, this study was the first to examine the moderating role of spirituality on the relations between young adolescents’ ToM and prosocial behavior across two different countries.

### Limitation, implications, and future direction

One of the main limitations in this study was the number of missing variables in the Iranian sample. Consequently, an unequal number of participants in culture and gender comparisons became another limitation of this study. Furthermore, we only examined the moderating role of two types of spirituality, existential and religious spirituality, on the relations between ToM and prosocial behaviors. Future studies are needed to investigate the moderating role of other types of spirituality on the relations among ToM and prosocial behavior in an equal sample size from both countries. In addition, the present study focused on the preferred gender identity of participants (male/female) – but given the changing definitions of gender and gender identities are conceived as more fluid – future studies should address this complexity (Andrews et al., 2021; Quenneville et al., 2022). Lastly, in the present study we used only one of the ToM measurements. Given the multifaceted mentalizing ability of ToM (Devine and Apperly, 2022), future studies should aim to more comprehensive measures of ToM to compare the direct and indirect effect of different ToM tasks on prosociality and spirituality among youth from different cultures.

### Conclusion

In sum, our study applied a complex ecological valid method to analyze the data to understand the complexity of ToM development and its relations with other social–emotional factors such as prosociality, spirituality, culture, and gender. Furthermore, our study suggests a complex nonlinear perspective as a theoretical framework for understanding social–emotional development. Our results indicate that Canadian adolescent females scored the highest in most of the social–emotional factors examined in this study, whereas Iranian adolescent males scored the lowest. Furthermore, Canadian participants generally showed higher scores in all factors than Iranian participants, except in religious well-being, which was higher in Iranian participants. These findings suggest that adolescent females are becoming the leaders in social–emotional development, whereas adolescent males need to continue to develop these abilities. Also, these abilities were more developed among Canadian youth compared to Iranian youth which may reflect cultural, political, and educational differences between the two countries.

**Table 7:** Moderating role of culture and duality on the relationship between ToM and prosocial behaviour.

Culture* duality	*b*	SE	*t*	*p*
Culture	−0.066	3.487	−0.019	0.984
Duality	3.368	1.197	2.812	0.005
ToM	0.452	0.596	0.758	0.449
ToM* culture	−0.062	0.196	−3.189	0.750
ToM* duality	−1.390	0.055	−2.499	0.013

**Table 8:** Moderating role of culture and existential well-being on the relationship between ToM and prosocial behaviour.

Culture* existential well-being	*b*	SE	*t*	*p*
Culture	−0.066	3.487	−0.019	0.984
Existential well-being	3.368	1.197	2.812	0.005
ToM	0.452	0.596	0.758	0.449
ToM* culture	−0.062	0.196	−3.189	0.750
ToM* existential well-being	−1.390	0.055	−2.499	0.013

## Data availability statement

The raw data supporting the conclusions of this article will be made available by the authors, without undue reservation.

## Ethics statement

The studies involving human participants were reviewed and approved by McGill University Research Ethics Board-II (REB#58-0615). Parents of the children provided their written informed consent and children provided verbal assent.

## Author contributions

NK: substantial contributions to the conception of the work; the acquisition, analysis, and interpretation of data for the work and drafting and revising it critically for important intellectual content. SB and VT: substantial contributions to the conception of the work and the acquisition, drafting the work and revising it critically for important intellectual content. All authors contributed to the article, approved the submitted version and agree to be accountable for all aspects of the work in ensuring that questions related to the accuracy or integrity of any part of the work are appropriately investigated and resolved.

## Funding

This research was supported in part by a grant from the Social Sciences and Humanities Research Council of Canada with grant no. 435–2015-0010 awarded to SB and VT. VT is Professor and Canada Research Chair in the Department of Educational and Counseling Psychology at McGill University. Her research is in the area of developmental psychology with an emphasis on social–emotional development. SB is a Professor in the Department of Graduate and Undergraduate Studies in Education at Brock University. Her research and teaching interests focus on social cognitive development in children and youth.

## Conflict of interest

The authors declare that the research was conducted in the absence of any commercial or financial relationships that could be construed as a potential conflict of interest.

## Publisher’s note

All claims expressed in this article are solely those of the authors and do not necessarily represent those of their affiliated organizations, or those of the publisher, the editors and the reviewers. Any product that may be evaluated in this article, or claim that may be made by its manufacturer, is not guaranteed or endorsed by the publisher.

## References

[ref1] Abrahamian E. (2021). Iran Between Two Revolutions. Princeton, New Jersey: Princeton University Press.

[ref2] Adams G. Kurtiş T. Salter P. S. Anderson S. L. (2012). “A cultural psychology of relationship: decolonizing science and practice” in Relationship Science: Integrating Evolutionary, Neuroscience, and Sociocultural Approaches. eds. Gillath O. Adams G. Kunkel A. (Washington, DC: American Psychological Association), 49–70.

[ref3] Ahrari A. Salmani F. Zeinali T. Izadi K. Yousefi A. Rahimi F.. (2022). Status of Iranian schools’ psycho-social environment: cultural adaptation and validation of the Persian version of the WHO profile to create child-friendly schools. BMC Public Health 22, 1–9. doi: 10.1186/s12889-022-14260-z 36195900 PMC9531853

[ref4] Aival-Naveh E. Rothschild-Yakar L. Kurman J. (2019). Keeping culture in mind: a systematic review and initial conceptualization of mentalizing from a cross-cultural perspective. Clin. Psychol. Sci. Pract. 26:e12300. doi: 10.1111/cpsp.12300

[ref5] Andrews K. Lariccia L. Talwar V. Bosacki S. (2021). Empathetic concern in emerging adolescents: the role of theory of mind and gender roles. J. Early Adolesc. 41, 1394–1424. doi: 10.1177/02724316211002258, PMID: 34712001 PMC8543568

[ref6] Antsaklis P. J. Michel A. N. (2006). Linear systems. Springer science & business media. J. Autism Dev. Disord. 41, 667–678. doi: 10.1007/s10803-010-1087-7

[ref7] Arab P. T. Maleki A. (2020). Iran’s secular shift: new survey reveals huge changes in religious beliefs. The Conversation UK. Available at: https://theconversation.com/irans-secular-shift-new-survey-reveals-huge-changes-in-religious-beliefs-145253

[ref8] Astington J. W. (2003). “Sometimes necessary, never sufficient: false belief understanding and social competence” in Individual Differences in Theory of Mind: Implications for Typical and Atypical Development. eds. Repacholi B. Slaughter V. (New York: Psychology Press), 13–38.

[ref9] Astington J. W. Pelletier J. Homer B. (2002). ToM and epistemological development: the relation between children’s second-order false-belief understanding and their ability to reason about evidence. New Ideas Psychol. Special Issue: Folk Epistemology 20, 131–144. doi: 10.1016/s0732-118x(02)00005-3

[ref10] Bagwell C. L. Newcomb A. F. Bukowski W. M. (1998). Preadolescent friendship and peer rejection as predictors of adult adjustment. Child Dev. 69, 140–153. doi: 10.1111/j.1467-8624.1998.tb06139.x, PMID: 9499563

[ref11] Bandura A. Walters R. H. (1977). Social Learning Theory (Vol. 1). Prentice Hall: Englewood cliffs.

[ref12] Baron-Cohen S. Wheelwright S. Hill J. Raste Y. Plumb I. (2001). The "Reading the mind in the eyes" test revised version: a study with normal adults, and adults with Asperger syndrome or high-functioning autism. J. Child Psychol. Psychiatry 42, 241–251. doi: 10.1111/1469-7610.00715 11280420

[ref13] Barrett R.. (2004). Liberating your soul: Accessing intuition and creativity. Available at: https://www.zenit.be/wp-content/uploads/2017/01/zenit-artikel-08Liberating-Your-Soul.pdf; http://www.soulfulliving.com/liberateyoursoul.htm.

[ref1005] Barrington L. (2012). Comparative Politics: Structures and Choices. Cengage Learning.

[ref1004] Beugelsdijk S. Welzel C. (2018). Dimensions and dynamics of national culture: Synthesizing Hofstede with Inglehart. J. Cross-cultural Psychol. 49, 1469–1505. doi: 10.1177/0022022118798505 PMC6191680 30369633

[ref14] Białecka-Pikul M. Kołodziejczyk A. Bosacki S. (2017). Advanced theory of mind in adolescence: do age, gender and friendship style play a role? J. Adolesc. 56, 145–156. doi: 10.1016/j.adolescence.2017.02.009, PMID: 28237631

[ref15] Blijd-Hoogewys E. M. van Geert P. L. (2017). Non-linearities in theory-of-mind development. Front. Psychol. 7:1970. doi: 10.3389/fpsyg.2016.01970 28101065 PMC5209372

[ref16] Boissevain J. Mitchell J. C. (Eds.) (2018). Network Analysis: Studies in Human Interaction. The Hague: Walter de Gruyter GmbH & Co KG.

[ref17] Bosacki S. L. (2000). Theory of mind and self-concept in preadolescents: links with gender and language. J. Educ. Psychol. 92, 709–717. doi: 10.1037/0022-0663.92.4.709

[ref18] Bosacki S. (2021). “Theory of mind and peer relationships in middle childhood and adolescence” in Theory of Mind in Middle Childhood and Adolescence. eds. Devine R. T. Lecce S. (London: Routledge), 142–168.

[ref19] Bosacki S. L. Moore C. (2004). Preschoolers' understanding of simple and complex emotions: links with gender and language. Sex Roles 50, 659–675. doi: 10.1023/B:SERS.0000027568.26966.27

[ref20] Bosacki S. Sitnik V. Dutcher K. Talwar V. (2018). Gratitude, social cognition, and well-being in emerging adolescents. J. Genet. Psychol. 179, 256–269. doi: 10.1080/00221325.2018.1499607, PMID: 30222076

[ref21] Bosacki S. L. Talwar V. Sitnik V. Pissoto F. M. Coccimiglio M. Quenneville S. (2020). Social cognition, self-perceptions, and social withdrawal in adolescents. Paedagogia Christiana 46, 63–91. doi: 10.12775/Pch.2020.019

[ref22] Braza F. Azurmendi A. Munoz J. M. Carreras M. R. Braza P. Gar~cıa A.. (2009). Social cognitive predictors of peer acceptance at age 5 and the moderating effects of gender. Br. J. Dev. Psychol. 27, 703–716. doi: 10.1348/026151008X360666, PMID: 19994576

[ref1006] Bufford R. K. Paloutzian R. F. Ellison C. W. (1991). Norms for the spiritual well-being scale. J. Psychol. Theol. 19, 56–70.

[ref23] Büssing A. Kerksieck P. Föller-Mancini A. Baumann K. (2012). Aspects of spirituality and ideals to help in adolescents from Christian academic high schools. Int. J. Child. Spiritual. 17, 99–116. doi: 10.1080/1364436X.2012.680882

[ref24] Caputi M. Schoenborn H. Walla P. (2018). Theory of mind and internalizing symptoms during middle childhood and early adolescence: the mediating role of coping strategies. Cogent Psychol. 5:1487270. doi: 10.1080/23311908.2018.1487270

[ref25] Carlo G. Knight G. P. McGinley M. Goodvin R. Roesch S. C. (2010). “The developmental relations between perspective taking and prosocial behaviors: a meta-analytic examination of the task-specificity hypothesis” in Self and Social Regulation: Social Interaction and the Development of Social Understanding and Executive Functions. eds. Sokol B. W. Müller U. Carpendale J. I. M. Young A. R. Iarocci G. (New York: Oxford University Press), 234–269.

[ref26] Carpendale J. I. M. Lewis C. (2015). “The development of social understanding” in Handbook of Child Psychology and Developmental Science: Cognitive Processes. eds. Liben L. S. Müller U. Lerner R. M. (Hoboken, New Jersey: John Wiley & Sons, Inc.), 381–424.

[ref27] Çelik S. B. Deniz M. E. (2008). A comparison of scouts' emotional intelligence levels with regards to age and gender variables: a cross-cultural study. İlköğretim Online 7, 376–383.

[ref28] Charman T. Ruffman T. Clements W. (2002). Is there a gender difference in development? Soc. Dev. 11, 1–10. doi: 10.1111/1467-9507.00183

[ref29] Chen X. Liu M. Bian Q. (2022). “Culture and children's social development” in The Wiley-Blackwell Handbook of Childhood Social Development. eds. Smith P. K. Hart C. H. (U. K: Blackwell Publishers), 241–259.

[ref30] Chen M. Zee M. Roorda D. L. (2021). Students' shyness and affective teacher-student relationships in upper elementary schools: a cross-cultural comparison. Learn. Individ. Differ. 86:101979. doi: 10.1016/j.lindif.2021.101979

[ref31] Chitakornkijsil P. (2010). Intercultural communication challenges and multinational organization communication. Int. J. Org. Innov. 3, 6–20.

[ref32] Coyne S. M. Linder J. R. Booth M. Keenan-Kroff S. Shawcroft J. E. Yang C. (2021). Princess power: longitudinal associations between engagement with princess culture in preschool and gender stereotypical behavior, body esteem, and hegemonic masculinity in early adolescence. Child Dev. 92, 2413–2430. doi: 10.1111/cdev.13633, PMID: 34287828

[ref33] Crocetti E. (2017). Identity formation in adolescence: the dynamic of forming and consolidating identity commitments. Child Dev. Perspect. 11, 145–150. doi: 10.1111/cdep.12226

[ref35] Denham S. A. (1986). Social cognition, prosocial behavior, and emotion in preschoolers: contextual validation. Child Dev. 57, 194–201. doi: 10.2307/1130651

[ref36] Derksen D. G. Hunsche M. C. Giroux M. E. Connolly D. A. Bernstein D. M. (2018). A systematic review of theory of mind’s precursors and functions. Z. Psychol. 226, 87–97. doi: 10.1027/2151-2604/a000325

[ref37] Devine R. T. Apperly I. A. (2022). Willing and able? Theory of mind, social motivation, and social competence in middle childhood and early adolescence. Dev. Sci. 25:e13137. doi: 10.1111/desc.13137, PMID: 34235829

[ref38] Diaz V. Farrar M. J. (2018). Do bilingual and monolingual preschoolers acquire false belief understanding similarly? The role of executive functioning and language. First Lang. 38, 382–398. doi: 10.1177/0142723717752741

[ref39] Dunfield K. A. (2014). A construct divided: Prosocial behavior as helping, sharing, and comforting subtypes. Front. Psychol. 5:958. doi: 10.3389/fpsyg.2014.00958, PMID: 25228893 PMC4151454

[ref40] Eisenberg N. (2003). “Prosocial behavior, empathy, and sympathy” in Well-being: Positive Development Across the Life Course. eds. Bornstein M. H. Davidson L. Keyes C. L. M. Moore K. A. (New York: Lawrence Erlbaum Associates Publishers), 253–265.

[ref41] Eisenberg N. Eggum N. D. Di Giunta L. (2010). Empathy-related responding: associations with prosocial behavior, aggression, and intergroup relations. Soc. Issues Policy Rev. 4, 143–180. doi: 10.1111/j.1751-2409.2010.01020.x, PMID: 21221410 PMC3017348

[ref42] Eisenberg N. Fabes R. A. (1998). Prosocial development. In Damon W. (Series Ed.) & Eisenberg N. (Vol. Ed.), Handbook of Child Psychology: Vol 3: Social, Emotional, and Personality Development (5th Edn., pp. 701–778). New York: Wiley.

[ref1003] Evason N. (2016). Family. Cultural Atlas. Available at: https://culturalatlas.sbs.com.au/iranian-culture/iranian-culture-family

[ref43] Fodor J. (1983). The Modularity of Mind. Cambridge, MA: The MIT Press.

[ref44] Gazelle H. Lundin J. K. S. Bosacki S. L. (2022). Theory of mind, gender, gains in friendships versus peer acceptance and anxious solitude from middle childhood through early adolescence. Soc. Dev. 1–21. doi: 10.1111/sode.12654

[ref45] Gershkoff-Stowe L. Thelen E. (2004). U-shaped changes in behavior: a dynamic systems perspective. J. Cogn. Dev. 5, 11–36. doi: 10.1207/s15327647jcd0501_2

[ref46] Gomez-Garibello C. Talwar V. (2015). Can you read my mind? Age as a moderator in the relationship between theory of mind and relational aggression. Int. J. Behav. Dev. 39, 552–559. doi: 10.1177/0165025415580805

[ref47] Grossmann T. (2017). The eyes as windows into other minds: an integrative perspective. Perspect. Psychol. Sci. 12, 107–121. doi: 10.1177/1745691616654457, PMID: 28073330

[ref48] Guajardo N. R. Parker J. Turley-Ames K. (2009). Associations among false belief understanding, counterfactual reasoning, and executive function. Br. J. Dev. Psychol. 27, 681–702. doi: 10.1348/026151008X357886, PMID: 19994575

[ref49] Gudykunst W. B. Ting-Toomey S. Chua E. (1988). Culture and Interpersonal Communication. Thousand Oaks, CA: Sage, 231.

[ref50] Hardy S. A. Carlo G. (2005a). Religiosity and prosocial behaviours in adolescence: the mediating role of prosocial values. J. Moral Educ. 34, 231–249. doi: 10.1080/03057240500127210

[ref51] Hardy S. A. Carlo G. (2005b). Identity as a source of moral motivation. Hum. Dev. 48, 232–256. doi: 10.1159/000086859

[ref52] Hardy S. A. Hendricks J. Nelson J. M. Schwadel P. (2022). Declines in religiousness dimensions across adolescence as predictors of religious deidentification in young adulthood. J. Res. Adolesc. 1–13. doi: 10.1111/jora.12786, PMID: 35860849

[ref53] Hastings P. D. Miller J. G. Troxel N. R. (2015). “Making good: the socialization of children's prosocial development” in Handbook of Socialization: Theory and Research. eds. Grusec J. E. Hastings P. D. (New York, NY: The Guilford Press), 637–660.

[ref54] Hay D. F. Cook K. V. (2007). “The transformation of prosocial behavior from infancy to childhood” in Socioemotional Development in the Toddler Years: Transitions and Transformations. eds. Brownell C. A. Kopp C. B. (New York, London: The Guilford Press), 100–131.

[ref55] Heeger D. (2000). Signals, linear systems, and convolution. Lecture notes (new York University, New York. Available at: http://www.cns.nyu.Edu/David/handouts/convolution.Pdf

[ref56] Hoffman M. L. (2000). Empathy and Moral Development: Implications for Caring and Justice. Cambridge, UK: Cambridge University Press.

[ref57] Hughes C. (2011). Social Understanding and Social Lives: From Toddlerhood Through to the Transition to School. London: Taylor & Francis.

[ref58] Hughes C. Ensor R. Marks A. (2011). Individual differences in false belief understanding are stable from 3 to 6 years of age and predict children’s mental state talk with school friends. J. Exp. Child Psychol. 108, 96–112. doi: 10.1016/j.jecp.2010.07.012, PMID: 20846667

[ref59] Hughes C. Leekam S. (2004). What are the links between theory of mind and social relations? Review, reflections and new directions for studies of typical and atypical development. Soc. Dev. 13, 590–619. doi: 10.1111/j.1467-9507.2004.00285.x

[ref60] Iglesias Gallego D. León-del-Barco B. Mendo-Lázaro S. Leyton-Román M. González-Bernal J. J. (2020). Modeling physical activity, mental health, and prosocial behavior in school-aged children: a gender perspective. Sustainability 12:4646. doi: 10.3390/su12114646

[ref61] Imuta K. Henry J. D. Slaughter V. Selcuk B. Ruffman T. (2016). Theory of mind and prosocial behavior in childhood: a meta-analytic review. Dev. Psychol. 52, 1192–1205. doi: 10.1037/dev0000140, PMID: 27337508

[ref62] Jans-Beken L. Lataster J. Peels D. Lechner L. Jacobs N. (2018). Gratitude, psychopathology and subjective well-being: results from a 7.5-month prospective general population study. J. Happiness Stud. 19, 1673–1689. doi: 10.1007/s10902-017-9893-7

[ref63] Khalili N. Bosacki S. Talwar V. (2022). Emerging adolescents’ perceptions of spirituality and gratitude. Int. J. Child. Spiritual. in press

[ref64] Kuhnert R. Begeer S. Fink E. de Rosnay M. (2017). Gender-differentiated effects of theory of mind, emotion understanding, and social preference on prosocial behavior development: a longitudinal study. J. Exp. Child Psychol. 154, 13–427. doi: 10.1016/j.jecp.2016.10.001, PMID: 27780091

[ref65] Lampridis E. Papastylianou D. (2017). Prosocial behavioural tendencies and orientation towards individualism–collectivism of Greek young adults. Int. J. Adolesc. Youth 22, 268–282. doi: 10.1080/02673843.2014.890114

[ref66] Lane J. D. Bowman L. C. (2021). How children’s social tendencies can shape their theory of mind development: access and attention to social information. Dev. Rev. 61:100977. doi: 10.1016/j.dr.2021.100977

[ref67] Leaper C. Farkas T. (2015). “The socialization of gender during childhood and adolescence” in Handbook of socialization: Theory and research. eds. Grusec J. E. Hastings P. D. (New York: The Guilford Press), 541–565.

[ref68] Lecce S. Devine R. T. (2021). “Social interaction in early and middle childhood” in The cognitive basis of social interaction across the lifespan. eds. Ferguson H. J. Bradford E. F. (London: Oxford University Press), 47–69.

[ref69] Leslie A. M. Knobe J. Cohen A. (2006). Acting intentionally and the side-effect effect: theory of mind and moral judgment. Psychol. Sci. 17, 421–427. doi: 10.1111/j.1467-9280.2006.01722.x, PMID: 16683930

[ref70] Longobardi E. Spataro P. Frigerio A. Rescorla L. (2016). Gender differences in the relationship between language and social competence in preschool children. Infant Behav. Dev. 43, 1–4. doi: 10.1016/j.infbeh.2016.03.001, PMID: 26974894

[ref71] Longobardi E. Spataro P. Rossi-Arnaud C. (2019). Direct and indirect associations of empathy, theory of mind, and language with prosocial behavior: gender differences in primary school children. J. Gen. Psychol. Res. Theor. Human Dev. 180, 266–279. doi: 10.1080/00221325.2019.1653817, PMID: 31456504

[ref72] Lowie W. (2012). “Dynamic systems theory approaches to second language acquisition” in The Encyclopedia of Applied Linguistics. ed. Chapelle C. (Chichester, West Sussex, UK: Wiley-Blackwell), 1–9.

[ref73] Luria G. Cnaan R. A. Boehm A. (2015). National culture and prosocial behaviors: results from 66 countries. Nonprofit Volunt. Sect. Q. 44, 1041–1065. doi: 10.1177/0899764014554456

[ref74] Martí-Vilar M. Serrano-Pastor L. Sala F. G. (2019). Emotional, cultural and cognitive variables of prosocial behaviour. Curr. Psychol. 38, 912–919. doi: 10.1007/s12144-019-0168-9

[ref75] Mason M. F. Morris M. W. (2010). Culture, attribution and automaticity: a social cognitive neuroscience view. Soc. Cogn. Affect. Neurosci. 5, 292–306. doi: 10.1093/scan/nsq034, PMID: 20460302 PMC2894680

[ref1002] Mayer A. F. Tra¨uble, B. (2013). Synchrony in the onset of mental state understanding across cultures? A study among children in Samoa. Int. J. Behav. Develop. 37, 21–28.

[ref76] McMahon S. D. Wernsman J. Parnes A. L. (2006). Understanding Prosocial behavior: the impact of empathy and gender among African American adolescents. J. Adolesc. Health 39, 135–137. doi: 10.1016/j.jadohealth.2005.10.008, PMID: 16781977

[ref77] Megías-Robles A. Gutiérrez-Cobo M. J. Cabello R. Gómez-Leal R. Baron-Cohen S. Fernández-Berrocal P. (2020). The ‘Reading the mind in the Eyes' test and emotional intelligence. R. Soc. Open Sci. 7:201305. doi: 10.1098/rsos.201305, PMID: 33047068 PMC7540806

[ref78] Mizokawa A. Hamana M. (2020). The relationship of theory of mind and maternal emotional expressiveness with aggressive behaviours in young Japanese children: a gender-differentiated effect. Infant Child Dev. 29:e2196. doi: 10.1002/icd.2196

[ref79] Mojdehi A. S. Shohoudi A. Talwar V. (2020). Children’s moral evaluations of different types of lies and parenting practices and across cultural contexts. Curr. Psychol. 41, 5420–5433. doi: 10.1007/s12144-020-01059-71-14

[ref80] Moore K. Gomez-Garibello C. Bosacki S. Talwar V. (2016). Children’s spiritual lives: the development of a children’s spirituality measure. Religions 7:95. doi: 10.3390/rel7080095

[ref81] Moore K. Talwar V. Bosacki S. (2012). Canadian children’s perceptions of spirituality: diverse voices. Int. J. Child. Spiritual. 17, 217–234. doi: 10.1080/1364436X.2012.742040

[ref82] Nabavi R. T. (2012). Bandura’s social learning theory and social cognitive learning theory. Theor. Dev. Psychol. 1:24.

[ref83] Nishimura S. Nevgi A. Tella S. (2008). Communication style and cultural features in high/low context communication cultures: a case study of Finland, Japan and India. Teoksessa a. Kallioniemi (toim.), Uudistuva ja kehittyvä ainedidaktiikka. Ainedidaktinen symposiumi 8, 783–796.

[ref84] Onder M. (2011). A preliminary cross-national test of competing theories of nonprofits: does culture matter? Int. Rev. Public Admin. 16, 71–90. doi: 10.1080/12264431.2011.10805186

[ref85] Pandya S. P. (2017). Spirituality, happiness, and psychological well-being in 13-to 15-year olds: a cross-country longitudinal RCT study. J. Pastoral Care Counsel. 71, 12–26. doi: 10.1177/1542305016687581 28279137

[ref87] Parboteeah K. P. Cullen J. B. Lim L. (2004). Formal volunteering: a cross-national test. J. World Bus. 39, 431–441. doi: 10.1016/j.jwb.2004.08.007

[ref88] Peterson C. C. Wellman H. M. (2019). Longitudinal theory of mind (ToM) development from preschool to adolescence with and without ToM delay. Child Dev. 90, 1917–1934. doi: 10.1111/cdev.13064, PMID: 29660808

[ref89] Post B. Wade N. (2009). Religion and spirituality in psychotherapy: a practice-friendly review of research. J. Clin. Psychol. 65, 131–146. doi: 10.1002/jclp.20563, PMID: 19132737

[ref90] Prevost M. Carrier M. E. Chowne G. Zelkowitz P. Joseph L. Gold I. (2014). The reading the mind in the eyes test: validation of a French version and exploration of cultural variations in a multi-ethnic city. Cogn. Neuropsychiatry 19, 189–204. doi: 10.1080/13546805.2013.823859, PMID: 23937473

[ref91] Quenneville S. Talwar V. Bosacki S. (2022). Teacher ratings and adolescent students’ perceived social behaviours and gender-role orientations. J. Gend. Stud. 31, 444–456. doi: 10.1080/09589236.2021.1988530

[ref92] Rabiei K. (2013). The development of sociology in the middle east: studying the obstacles since the 20th century. J. Iranian Social Stud. 6, 77–101.

[ref93] Razza R. A. Blair C. (2009). Associations among false belief understanding, executive function, and social competence: a longitudinal analysis. J. Appl. Dev. Psychol. 30, 332–343. doi: 10.1016/j.appdev.2008.12.020, PMID: 20161159 PMC2735755

[ref94] Renouf A. Brendgen M. Séguin J. R. Vitaro F. Boivin M. Dionne G.. (2010). Interactive links between theory of mind, peer victimization, and reactive and proactive aggres- Sion. J. Abnorm. Child Psychol. 38, 1109–1123. doi: 10.1007/s10802-010-9432-z, PMID: 20544385 PMC3283569

[ref95] Rezapour M. Khanjani N. Mirzai M. (2019). Exploring associations between school environment and bullying in Iran: multilevel contextual effects modeling. Child Youth Serv. Rev. 99, 54–63. doi: 10.1016/j.childyouth.2019.01.036

[ref96] Ringov D. Zollo M. (2007). The impact of national culture on corporate social performance. Corp. Gov. 7, 476–485. doi: 10.1108/14720700710820551

[ref97] Roberts W. Strayer J. (1996). Empathy, emotional expressiveness, and prosocial behavior. Child Dev. 67, 449–470. doi: 10.2307/1131826

[ref99] Saunders C. Fernyhough C. (2016). The medieval mind. The British Psychological Society. Available at: https://www.bps.org.uk/psychologist/medieval-mind.

[ref100] Schmidtmann G. Logan A. J. Carbon C. C. Loong J. T. Gold I. (2020). In the blink of an eye: Reading mental states from briefly presented eye regions. i-Perception 11:2041669520961116. doi: 10.1177/2041669520961116, PMID: 33088473 PMC7543157

[ref101] Schonert-Reichl K. A. Hymel S. (2007). Educating the heart as well as the mind social and emotional learning for school and life success. Educ. Can. 47, 20–25.

[ref102] Shahaeian A. (2015). Sibling, family, and social influences on children’s theory of mind understanding: new evidence from diverse intracultural samples. J. Cross-Cult. Psychol. 46, 805–820. doi: 10.1177/0022022115583897

[ref103] Shakoor S. Jaffee S. R. Bowes L. Ouellet-Morin I. Andreou P. Happé F.. (2011). A prospective longitudinal study of children’s theory of mind and adolescent involvement in bullying. J. Child Psychol. Psychiatry 53, 254–261. doi: 10.1111/j.1469-7610.2011.02488.x, PMID: 22081896 PMC3272094

[ref104] Shohoudi Mojdehi A. Leduc K. Shohoudi Mojdehi A. Talwar V. (2019). Examining cross-cultural differences in youth's moral perceptions of cyberbullying. Cyberpsychol. Behav. Soc. Netw. 22, 243–248. doi: 10.1089/cyber.2018.0339, PMID: 30848665

[ref105] Shohoudi Mojdehi A. Shohoudi A. Talwar V. (2020). Moral evaluations of modest statements in light of maternal disciplinary method and cultures. J. Moral Educ. 50, 494–511. doi: 10.1080/03057240.2020.1781602

[ref106] Slaughter V. Imuta K. Peterson C. C. Henry J. D. (2015). Meta-analysis of theory of mind and peer popularity in the preschool and early school years. Child Dev. 86, 1159–1174. doi: 10.1111/cdev.12372, PMID: 25874384

[ref1001] Smith J. R. McSweeney A. (2007). Charitable giving: The effectiveness of a revised theory of planned behaviour model in predicting donating intentions and behaviour. J. Commun. App. Soc. Psychol. 17, 363–386. doi: 10.1002/casp.906

[ref107] Souza D. D. H. Suárez S. Koenig M. A. (2021). Selective trust and theory of mind in Brazilian children: effects of culture or socioeconomic background? J. Cogn. Dev. 22, 169–184. doi: 10.1080/15248372.2020.1867553

[ref108] Spilka B. Hood Jr. R. W. Hunsberger B. Gorsuch R. (2003). The psychology of religion: An empirical approach (3rd ed.). New York: Guilford.

[ref110] Sy S. R. DeMeis D. K. Scheinfield R. E. (2003). Pre-school children's understanding of the emotional consequences for failures to act prosocially. Br. J. Dev. Psychol. 21, 259–272. doi: 10.1348/026151003765264075

[ref111] Tabassum S. Pereira F. S. Fernandes S. Gama J. (2018). Social network analysis: an overview. Wiley interdisciplinary reviews. Data Min. Knowl. Disc. 8:e 1256. doi: 10.1002/widm.1256

[ref112] Testoni I. Pesci S. De Vincenzo C. Dal Corso L. Zamperini A. (2019). Work and spirituality among people with Asperger syndrome: an exploratory study. J. Disability Religion 23, 178–196. doi: 10.1080/23312521.2019.1580174

[ref113] Underwood B. Moore B. (1982). Perspective-taking and altruism. Psychol. Bull. 91, 143–173. doi: 10.1037/0033-2909.91.1.143

[ref114] Van der Graaff J. Carlo G. Crocetti E. Koot H. M. Branje S. (2018). Prosocial behavior in adolescence: gender differences in development and links with empathy. J. Youth Adolesc. 47, 1086–1099. doi: 10.1007/s10964-017-0786-1, PMID: 29185207 PMC5878203

[ref115] Van Elk M. Aleman A. (2017). Brain mechanisms in religion and spirituality: an integrative predictive processing framework. Neurosci. Biobehav. Rev. 73, 359–378. doi: 10.1016/j.neubiorev.2016.12.031, PMID: 28041787

[ref116] Van Geert P. (2003). “Dynamic systems approaches and modeling of developmental processes’,’ in Handbook of Developmental Psychology. eds. J. Valsiner and K. J. Connolly (London: Sage), 640–672.

[ref117] Vittengl J. R. (2018). A lonely search?: risk for depression when spirituality exceeds religiosity. J. Nerv. Ment. Dis. 206, 386–389. doi: 10.1097/NMD.0000000000000815 29652773

[ref118] Wainryb C. Brehl B. A. (2006). “I thought she knew that would hurt my feelings: developing psychological knowledge and moral thinking” in Advances in Child Development and Behavior. ed. Kail R. V., 131–171. 17120804 10.1016/s0065-2407(06)80006-6

[ref119] Wang S. Andrews G. Pendergast D. Neumann D. Chen Y. Shum D. H. (2022). A cross-cultural study of theory of mind using strange stories in school-sged children from Australia and mainland China. J. Cogn. Dev. 23, 40–63. doi: 10.1080/15248372.2021.1974445

[ref121] Warneken F. Tomasello M. (2006). Altruistic helping in human infants and young chimpanzees. Science 311, 1301–1303. https://doi:10.1126/science.1121448. doi: 10.1126/science.1121448, PMID: 16513986

[ref122] Warneken F. Tomasello M. (2007). Helping and cooperation at 14 months of age. Infancy 11, 271–294. doi: 10.1111/j.1532-7078.2007.tb00227.x, PMID: 33412734

[ref123] Weller D. Lagattuta K. H. (2014). Children's judgments about prosocial decisions and emotions: gender of the helper and recipient matters. Child Dev. 85, 2011–2028. doi: 10.1111/cdev.12238, PMID: 24611809

